# Supplementary material for: Enhanced TARP-γ8-PSD-95 coupling in excitatory neurons contributes to the rapid antidepressant-like action of ketamine in male mice
Source: Nat Commun. 2023 Dec 2;14:7971. doi: 10.1038/s41467-023-42780-8 (PMC10693574; doi:10.1038/s41467-023-42780-8)

Supplementary information

Enhanced TARP-γ8-PSD-95 coupling in excitatory neurons contributes to the rapid antidepressant-like action of ketamine in male mice

Shi-Ge Xue[1,5], Jin-Gang He[1,2,3,4,5], Ling-Li Lu[1], Shi-Jie Song[1], Mei-Mei Chen[1], Fang Wang[1,2,3,4*] and Jian-Guo Chen[1,2,3,4*]

[1]State Key Laboratory for Diagnosis and Treatment of Severe Zoonotic Infectious Diseases, Department of Pharmacology, School of Basic Medicine, Tongji Medical College, Huazhong University of Science and Technology, Wuhan, China. [2]The Key Laboratory for Drug Target Researches and Pharmacodynamic Evaluation of Hubei Province, Wuhan, China. [3]The Research Center for Depression, Tongji Medical College, Huazhong University of Science, 430030, Wuhan, China. [4]Key Laboratory of Neurological Diseases (HUST), Ministry of Education of China, Wuhan, China. [5]These authors contributed equally: Shi-Ge Xue, Jin-Gang He.

*Correspondence: J.-G. Chen, chenj@mails.tjmu.edu.cn or F. Wang, wangfanghust@hust.edu.cn

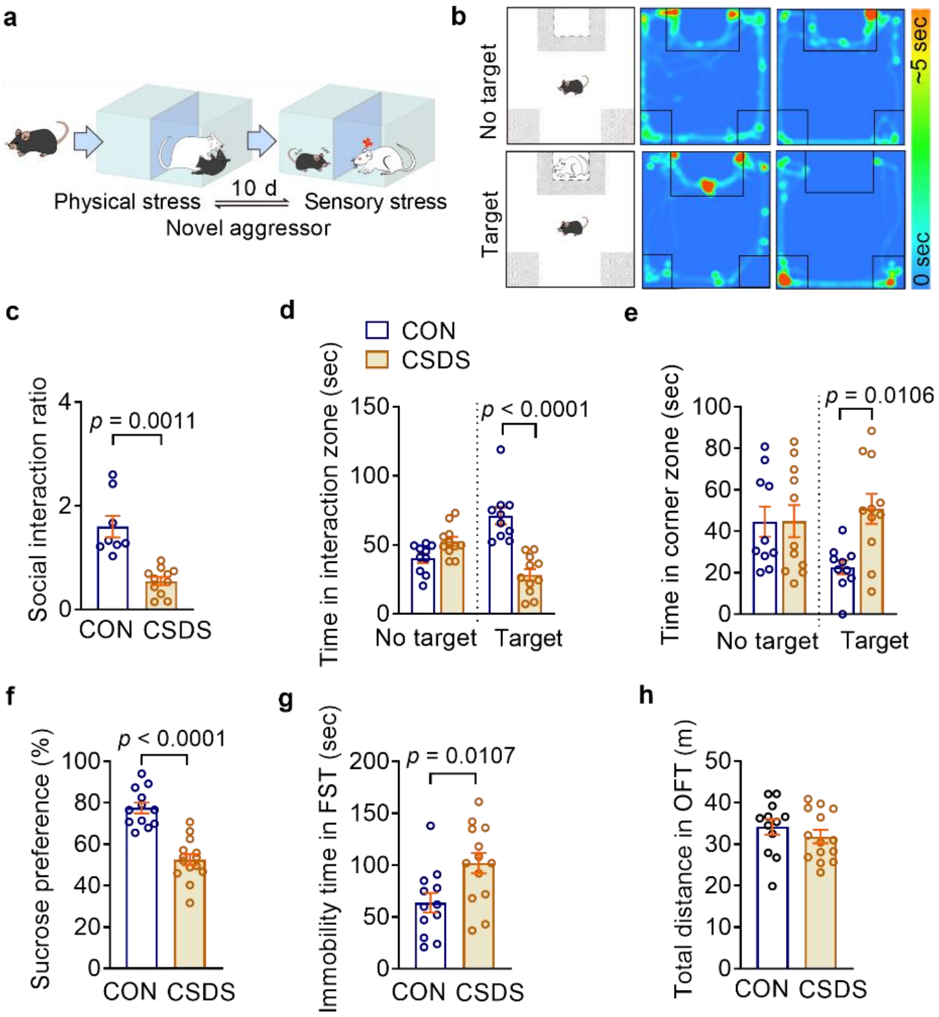

Supplementary Fig. 1 CSDS induces depressive-like behaviors in mice.

a Schematic diagram of CSDS. **b** Representative heatmap of social interaction data. **c** Social interaction ratio of SIT (n = 8 in CON, n = 11 in CSDS). **d, e** Time in the interaction zone of SIT **(d)** and time in the corner zones of SIT **(e)** (n = 10 in CON, n = 11 in CSDS). **f** Preference for sucrose in the SPT (n = 12 in CON, n = 14 in CSDS). **g** Immobility time in the FST (n = 12 in CON, n = 14 in CSDS). **h** Total distance in the OFT (n = 12 in CON, n = 14 in CSDS). Comparisons were performed by unpaired, two-tailed t test in **(c, f-h)** and by Two-way ANOVA analysis followed by Bonferroni's multiple comparisons test in **(d, e)**. Data are presented as mean ± SEM. All numbers (n) are biologically independent experiments. Source data are provided as a Source Data file.

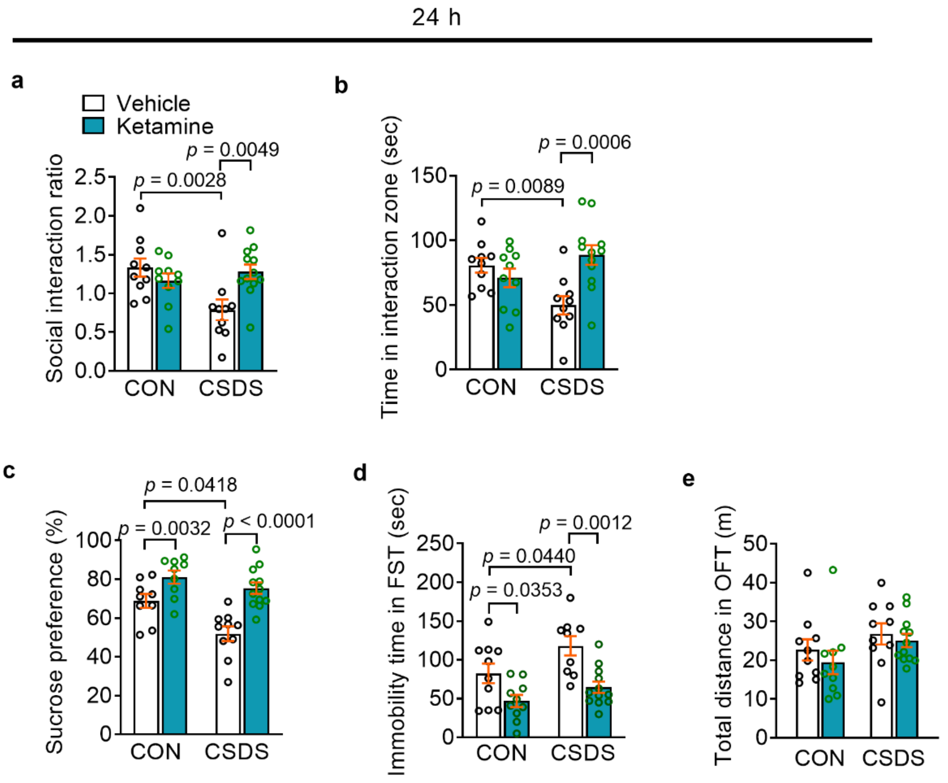

Supplementary Fig. 2 Ketamine exerts antidepressant effects 24 h after administration in CSDS model.

a Social interaction ratio of SIT (*n* = 10, 10, 10 and 12 in CON-Vehicle, CON-Ketamine, CSDS-Vehicle and CSDS-Ketamine, respectively). **b** Time in the interaction zone of SIT (*n* = 10, 10, 10 and 12 in CON-Vehicle, CON-Ketamine, CSDS-Vehicle and CSDS-Ketamine, respectively). **c** Preference for sucrose in the SPT (*n* = 9, 9, 10 and 12 in CON-Vehicle, CON-Ketamine, CSDS-Vehicle and CSDS-Ketamine, respectively). **d** Immobility time in the FST (*n* = 10, 10, 9 and 12 in CON-Vehicle, CON-Ketamine, CSDS-Vehicle and CSDS-Ketamine, respectively). **e** Total distance in the OFT (*n* = 10, 10, 10 and 12 in CON-Vehicle, CON-Ketamine, CSDS-Vehicle and CSDS-Ketamine, respectively). Comparisons were performed by Two-way ANOVA analysis followed by Bonferroni's multiple comparisons test. Data are presented as mean ± SEM. All numbers (*n*) are biologically independent experiments. Source data are provided as a Source Data file.

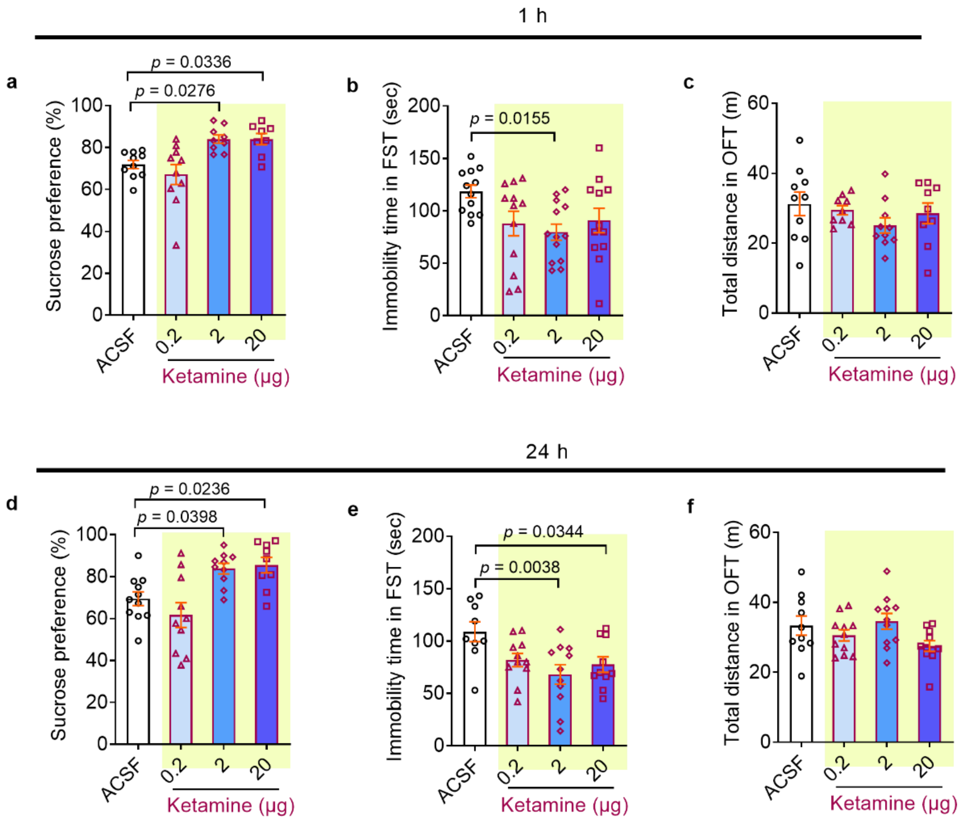

Supplementary Fig. 3 Intral-ventral hippocampus injection of ketamine exerts antidepressant effects.

a Preference for sucrose in the SPT (n = 10, 10, 9 and 9 in ACSF, 0.2 μg-Ketamine, 2 μg-Ketamine and 20 μg-Ketamine, respectively). **b** Immobility time in the FST (n = 12, 12, 13 and 12 in ACSF, 0.2 μg-Ketamine, 2 μg-Ketamine and 20 μg-Ketamine, respectively). **c** Total distance in the OFT (n = 10, 9, 10 and 9 in ACSF, 0.2 μg-Ketamine, 2 μg-Ketamine and 20 μg-Ketamine, respectively). **d** Preference for sucrose in the SPT (n = 11, 10, 10 and 9 in ACSF, 0.2 μg-Ketamine, 2 μg-Ketamine and 20 μg-Ketamine, respectively). **e** Immobility time in the FST (n = 9, 11, 11 and 10 in ACSF, 0.2 μg-Ketamine, 2 μg-Ketamine and 20 μg-Ketamine, respectively). **f** Total distance in the OFT (n = 10, 11, 11 and 11 in ACSF, 0.2 μg-Ketamine, 2 μg-Ketamine and 20 μg-Ketamine, respectively). Comparisons were performed by One-way ANOVA analysis followed by Dunnett's multiple comparisons test. Data are presented as mean ± SEM. All numbers (n) are biologically independent experiments. Source data are provided as a Source Data file.

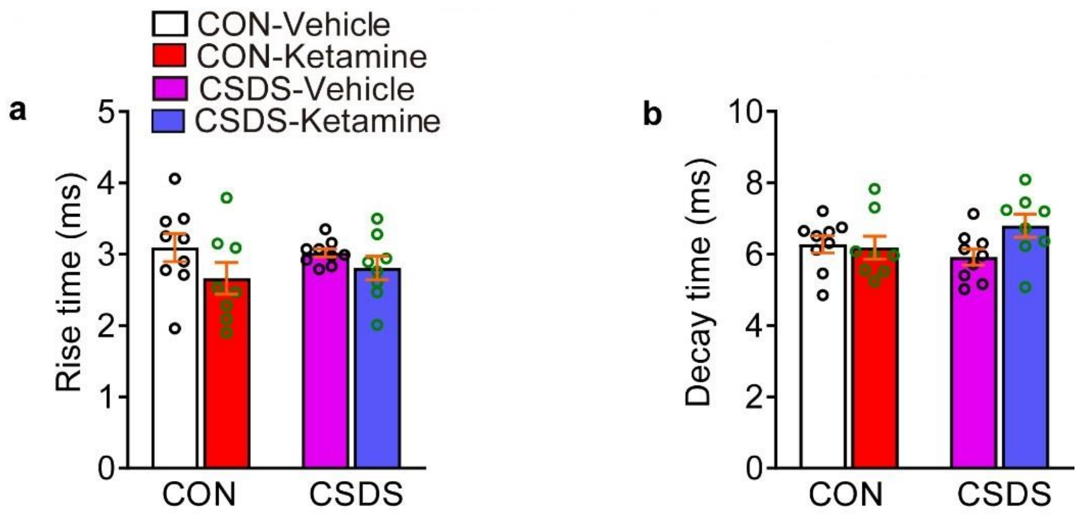

Supplementary Fig. 4 The kinetic properties of AMPAR are not affected by Ketamine.

a, b The rise time **(a)** and decay time (**b**) of AMPARs-mediated mEPSC in the ventral hippocampal CA1 neurons between different groups (*n* = 9 cells from 4 mice in CON-Vehicle, *n* = 8 cells from 3 mice in CON-Ketamine, *n* = 9 cells from 3 mice in CSDS-Vehicle, *n* = 8 cells from 4 mice in CSDS-Ketamine). Comparisons were performed by Two-way ANOVA analysis followed by Bonferroni's multiple comparisons test. Data are presented as mean ± SEM. All numbers (*n*) are biologically independent experiments. Source data are provided as a Source Data file.

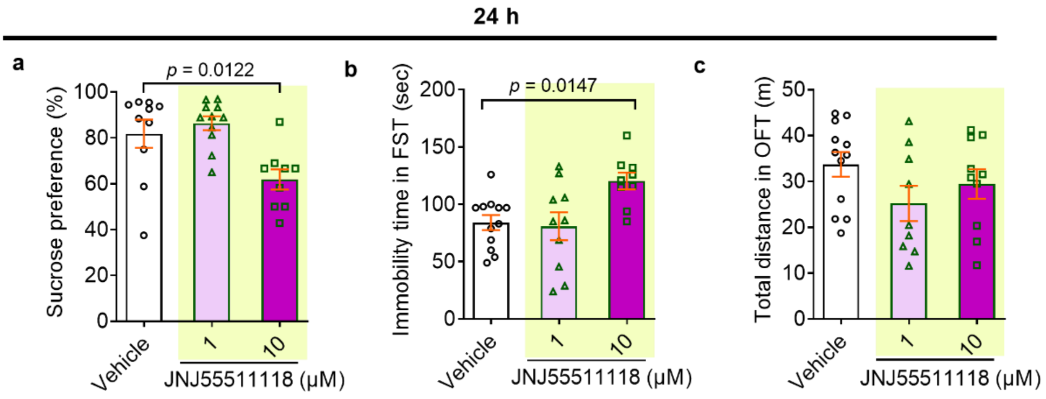

Supplementary Fig. 5 Intra-ventral hippocampus injection of JNJ55511118 induces depression-like behaviors in male mice.

a Preference for sucrose in the SPT (*n* = 10, 11, and 9 in Vehicle, 1 μM-Ketamine and 10 μM-Ketamine, respectively). **b** Immobility time in the FST (*n* = 12, 10, and 9 in Vehicle, 1 μM-Ketamine and 10 μM-Ketamine, respectively). **c** Total distance in the OFT (*n* = 12, 9, and 10 in Vehicle, 1 μM-Ketamine and 10 μM-Ketamine, respectively). Comparisons were performed by One-way ANOVA analysis followed by Dunnett's multiple comparisons test. Data are presented as mean ± SEM. All numbers (*n*) are biologically independent experiments. Source data are provided as a Source Data file.

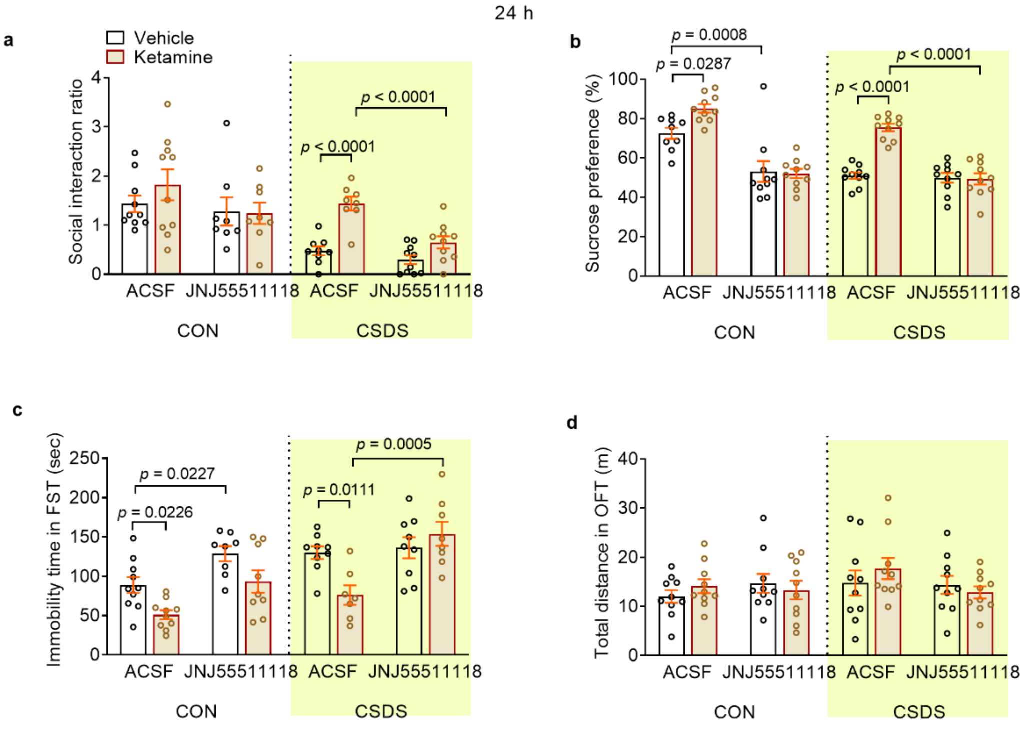

Supplementary Fig. 6 Pre-administration of JNJ55511118 into the ventral hippocampus abolishes the antidepressant effects of ketamine in CSDS model.

a Social interaction ratio of SIT (n = 10, 10, 8, 8, 9, 8, 10 and 10 in CON-ACSF-Vehicle, CON-ACSF-Ketamine, CON-JNJ55511118-Vehicle, CON-JNJ55511118-Ketamine, CSDS-ACSF-Vehicle, CSDS-ACSF-Ketamine, CSDS-JNJ55511118-Vehicle and CSDS-JNJ55511118-Ketamine, respectively). **b** Preference for sucrose in the SPT (n = 9, 10, 10, 10, 10, 10, 10 and 10 in CON-ACSF-Vehicle, CON-ACSF-Ketamine, CON-JNJ55511118-Vehicle, CON-JNJ55511118-Ketamine, CSDS-ACSF-Vehicle, CSDS-ACSF-Ketamine, CSDS-JNJ55511118-Vehicle and CSDS-JNJ55511118-Ketamine, respectively). **c** Immobility time in the FST (n = 10, 10, 8, 9, 9, 7, 9 and 8 in CON-ACSF-Vehicle, CON-ACSF-Ketamine, CON-JNJ55511118-Vehicle, CON-JNJ55511118-Ketamine, CSDS-ACSF-Vehicle, CSDS-ACSF-Ketamine, CSDS-JNJ55511118-Vehicle and CSDS-JNJ55511118-Ketamine, respectively). **d** Total distance travelled in the OFT (n = 10 mice per group). Comparisons were performed by Two-way ANOVA analysis followed by Bonferroni's multiple comparisons test. Data are presented as mean ± SEM. All numbers (n) are biologically independent experiments. Source data are provided as a Source Data file.

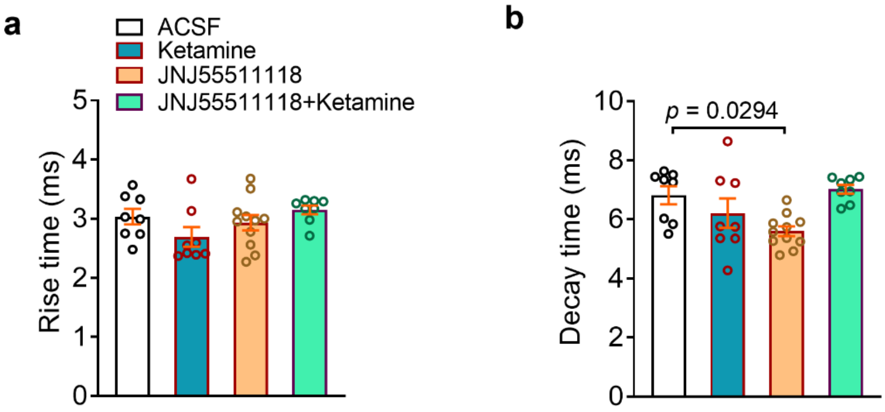

Supplementary Fig. 7 Influence of ketamine and JNJ55511118 on kinetic properties of AMPARs.

a No changes were observed in the rise time of AMPARs-mediated mEPSC in the ventral hippocampal CA1 neurons between different groups (n = 8 cells from 4 mice in ACSF, n = 8 cells from 4 mice in Ketamine, n = 11 cells from 5 mice in JNJ55511118, n = 8 cells from 4 mice in JNJ55511118-Ketamine). **b** JNJ55511118 reduced the decay time of AMPARs-mediated mEPSC in the ventral hippocampal CA1 neurons (n = 8 cells from 4 mice in ACSF, n = 8 cells from 4 mice in Ketamine, n = 11 cells from 5 mice in JNJ55511118, n = 8 cells from 4 mice in JNJ55511118-Ketamine). Comparisons were performed by One-way ANOVA analysis followed by Bonferroni's multiple comparisons test. Data are presented as mean ± SEM. All numbers (n) are biologically independent experiments. Source data are provided as a Source Data file.

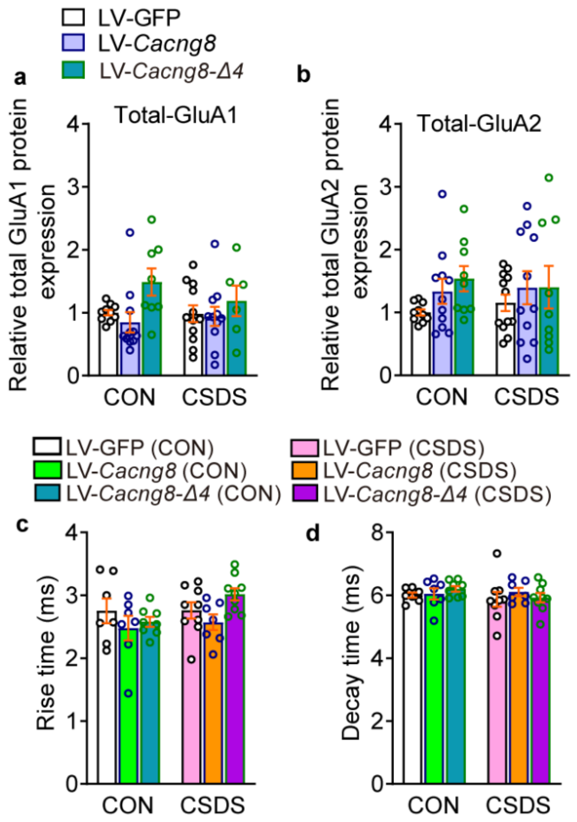

Supplementary Fig. 8 The expression level of total AMPARs is not affected by CSDS or overexpression of TARP-γ8/TARP-γ8-Δ4 in the ventral hippocampus.

a, b Expression of total GluA1 **(a)** (n = 10, 12, 11, 11, 8 and 6 in CON-LV-GFP, CSDS-LV-GFP, CON-LV-*Cacng8*, CSDS-LV-*Cacng8*, CON-LV-*Cacng8-Δ4* and CSDS-LV-*Cacng8-Δ4*, respectively) and GluA2 **(b)** (n = 10, 12, 11, 11, 9 and 9 in CON-LV-GFP, CSDS-LV-GFP, CON-LV-*Cacng8*, CSDS-LV-*Cacng8*, CON-LV-*Cacng8-Δ4* and CSDS-LV-*Cacng8-Δ4*, respectively) protein level in the ventral hippocampus. **c, d** No change was observed in the rise time **(c)** or decay time **(d)** of AMPARs-mediated mEPSC in the ventral hippocampal CA1 neurons between different groups (n = 7 cells from 5 mice in CON-LV-GFP, n = 9 cells from 6 mice in CSDS-LV-GFP, n = 7 cells from 6 mice in CON-LV-*Cacng8*, n = 7 cells from 5 mice in CSDS-LV-*Cacng8*, n = 8 cells from 6 mice in CON-LV-*Cacng8-Δ4* and n = 9 cells from 6 mice in CSDS-LV-*Cacng8-Δ4*). Comparisons were performed by Two-way ANOVA analysis followed by Bonferroni's multiple comparisons test. Data are presented as mean ± SEM. All numbers (n) are biologically independent experiments. Source data are provided as a Source Data file.

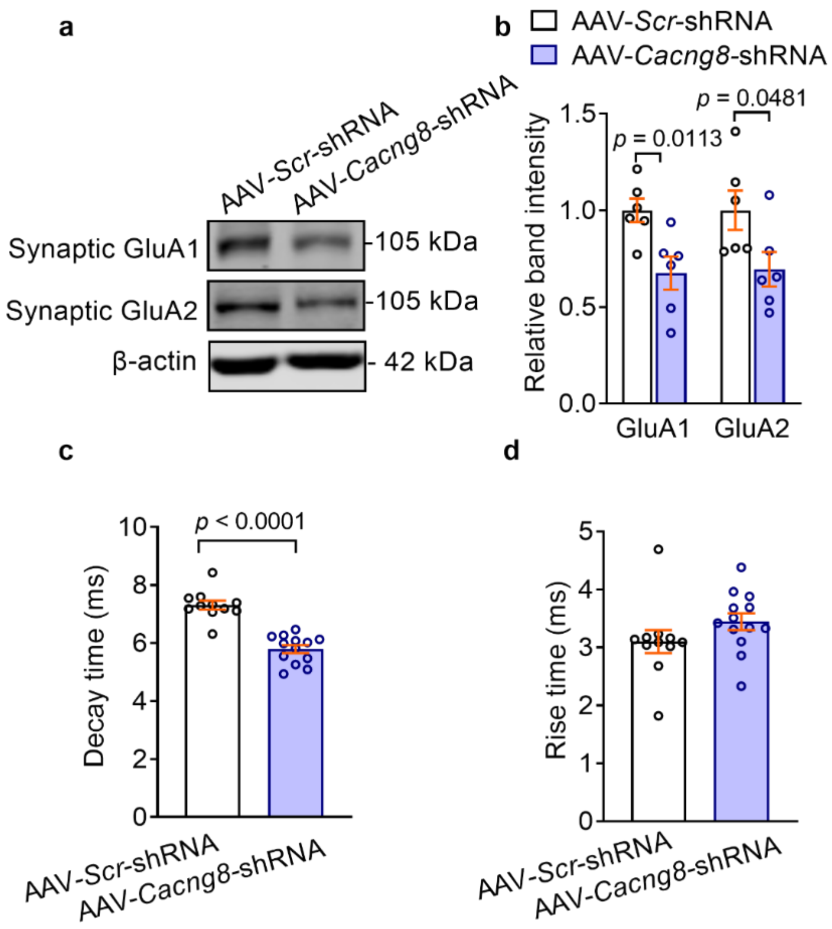

Supplementary Fig. 9 Knockdown of TARP-γ8 in the ventral hippocampus decreases AMPAR subunits expression in the synaptic compartment.

a, b The representative image of western blot **(a)**, and the quantification **(b)** of the level of GluA1 and GluA2 in the synaptic compartment in the ventral hippocampus of TARP-γ8 knockdown mice (n = 6 samples per group). **c, d** The decay time **(c)** and the rise time **(d)** of AMPARs-mediated mEPSC. (n = 11 cells from 4 mice in AAV-*Scr*-shRNA, n = 13 cells from 6 mice in AAV-*Cacng8*-shRNA). Comparisons were performed by unpaired, two-tailed t test. Data are presented as mean ± SEM. All numbers (n) are biologically independent experiments. Uncropped blots of **(a)** are supplied at the end. Source data are provided as a Source Data file.

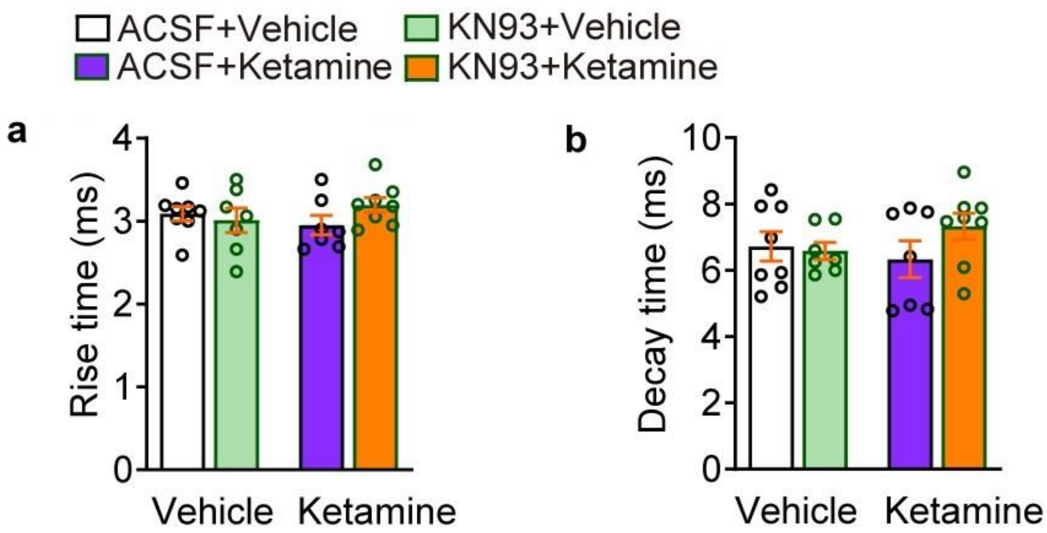

Supplementary Fig. 10 Influence of ketamine and KN93 on kinetic properties of AMPARs.

a, b The rise time **(a)** and decay time (**b**) of AMPARs-mediated mEPSC in the ventral hippocampal CA1 neurons among different groups (*n* = 8 cells from 4 mice in Vehicle-ACSF, *n* = 7 cells from 4 mice in Vehicle-KN93, *n* = 7 cells from 4 mice in Ketamine-ACSF and *n* = 8 cells from 4 mice in Ketamine-KN93). Comparisons were performed by Two-way ANOVA analysis followed by Bonferroni's multiple comparisons test. Data are presented as mean ± SEM. All numbers (*n*) are biologically independent experiments. Source data are provided as a Source Data file.

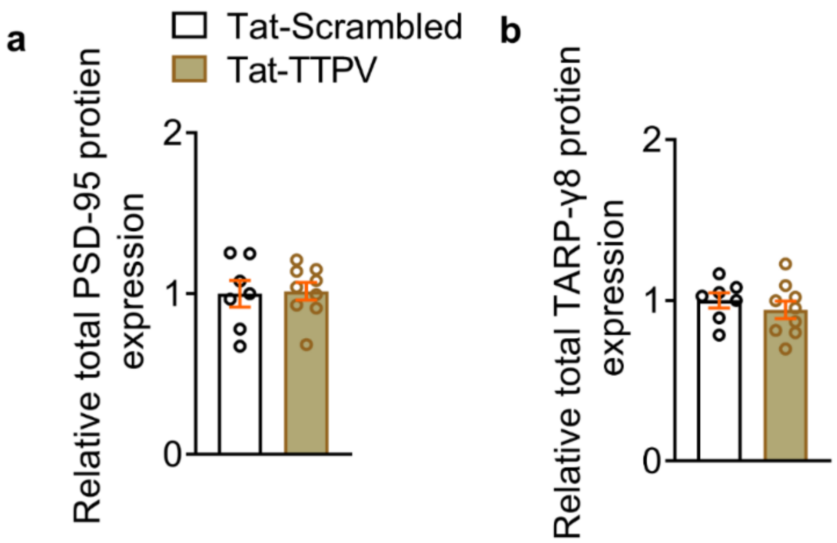

Supplementary Fig. 11 Influence of Tat-TTPV on expression of PSD-95 and TARP-γ8.

a Total expression of PSD-95 was not changed between different groups (*n* = 7 in Tat-Scrambled, *n* = 9 in Tat-TTPV). **b** Total expression of TARP-γ8 protein was not changed between different groups (*n* = 7 in Tat-Scrambled, *n* = 9 in Tat-TTPV). Comparisons were performed by unpaired, two-tailed *t* test. Data are presented as mean ± SEM. All numbers (*n*) are biologically independent experiments. Source data are provided as a Source Data file.

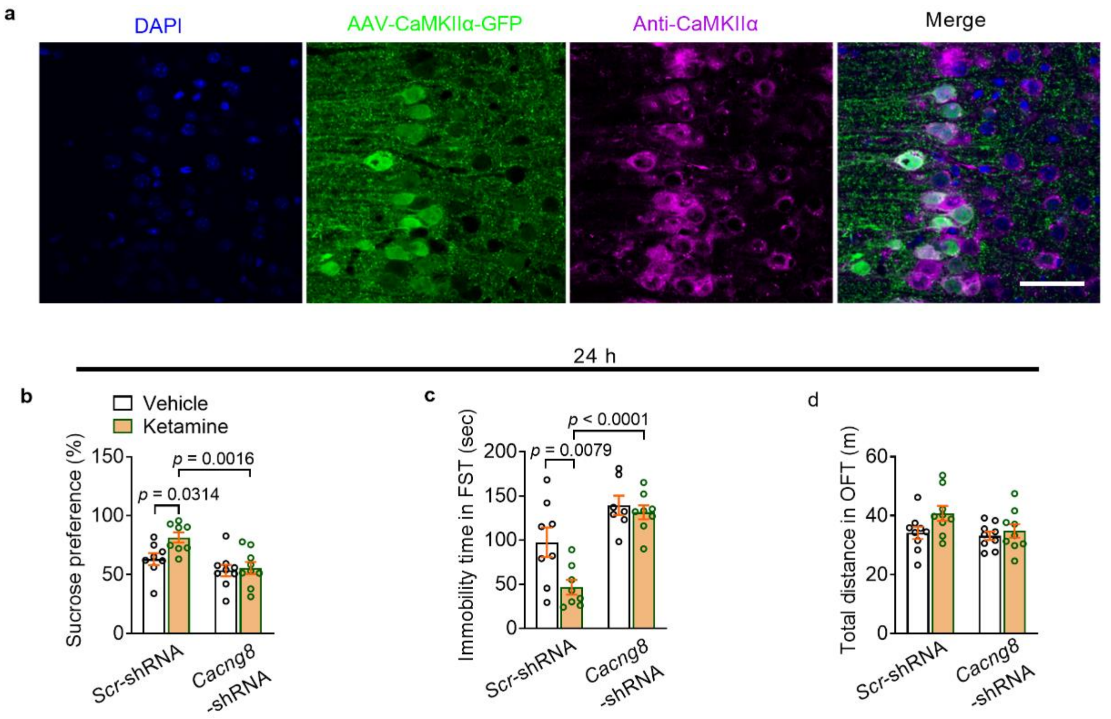

Supplementary Fig. 12 Knockdown of TARP-γ8 in excitatory neurons in the ventral hippocampus blocks the antidepressant effects of ketamine.

a Immunofluorescence verified that virus-infected neurons overlap with CaMKIIα-expressing neurons. DAPI (blue), AAV-CaMKIIα-GFP (green), Anti-CaMKIIα (purple). Scale bar = 50 μm. Experiments were repeated independently 3 times with similar results. **b** Preference for sucrose in the SPT (n = 8, 9, 8 and 9 in Vehicle-*Scr*-shRNA, Vehicle-*Cacng8*-shRNA, Ketamine-*Scr*-shRNA and Ketamine-*Cacng8*-shRNA, respectively). **c** Immobility time in the FST (n = 8, 7, 8 and 8 in Vehicle-*Scr*-shRNA, Vehicle-*Cacng8*-shRNA, Ketamine-*Scr*-shRNA and Ketamine-*Cacng8*-shRNA, respectively). **d** Total distance in the OFT (n = 9 samples per group). Comparisons were performed by Two-way ANOVA analysis followed by Bonferroni's multiple comparisons test. Data are presented as mean ± SEM. All numbers (n) are biologically independent experiments. Source data are provided as a Source Data file.

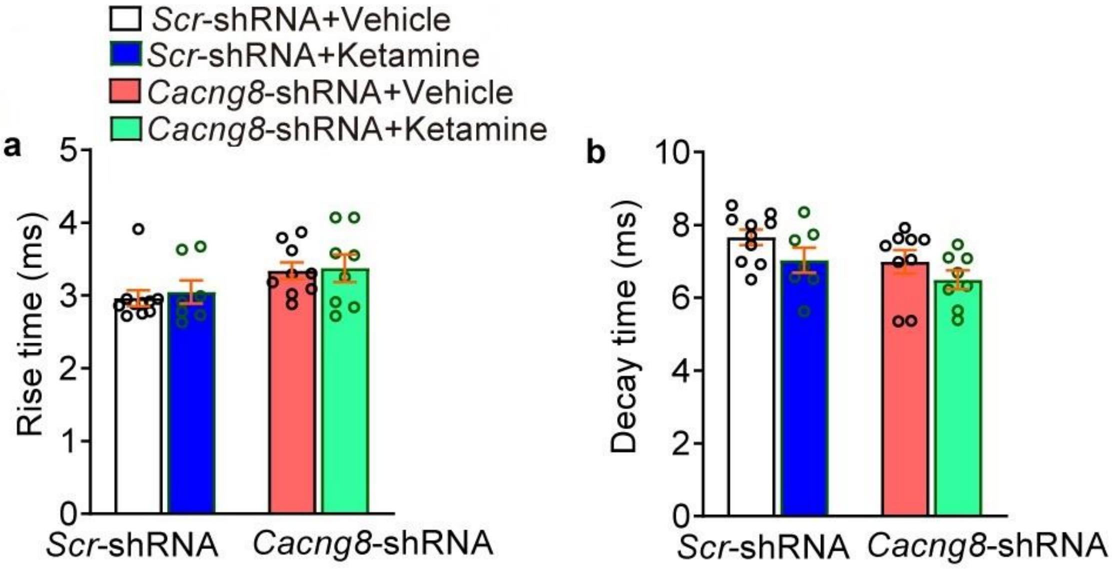

Supplementary Fig. 13 Influence of ketamine and knockdown of TARP-γ8 in excitatory neuron on kinetic properties of AMPARs.

a, b No change was observed in rise time (**a**) or decay time (**b**) between different groups (n = 10 cells from 4 mice in Vehicle-*Scr*-shRNA, n = 9 cells from 4 mice in Vehicle-*Cacng8*-shRNA, n = 7 cells from 4 mice in Ketamine-*Scr*-shRNA and n = 8 cells from 4 mice in Ketamine-*Cacng8*-shRNA). Comparisons were performed by Two-way ANOVA analysis followed by Bonferroni's multiple comparisons test. Data are presented as mean ± SEM. All numbers (n) are biologically independent experiments. Source data are provided as a Source Data file.

Uncropped scans of western blot:

Supplementary Figure 9a

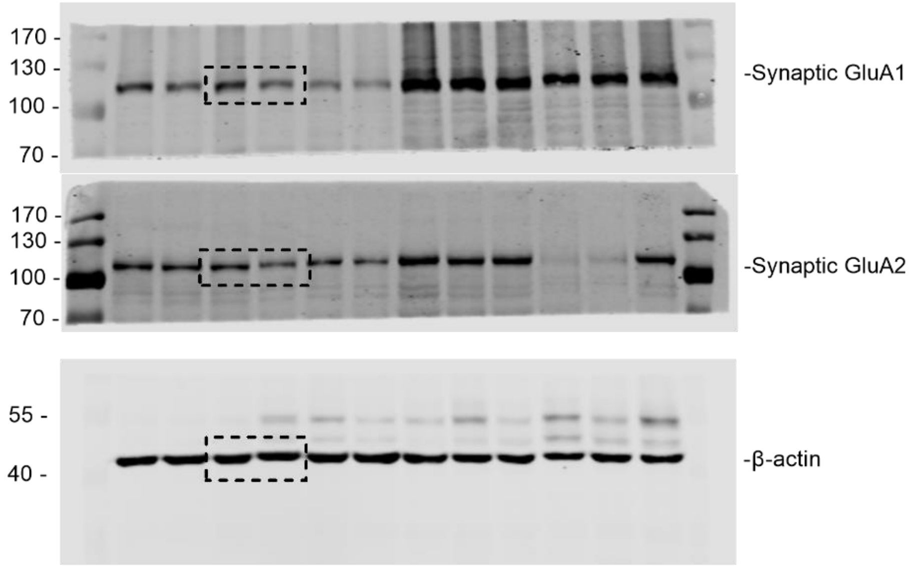

Supplement: Supplementary file 1 — Supplementary Information [file 41467_2023_42780_MOESM1_ESM.pdf]
