# Supplementary material for: Enhanced TARP-γ8-PSD-95 coupling in excitatory neurons contributes to the rapid antidepressant-like action of ketamine in male mice
Source: Nat Commun. 2023 Dec 2;14:7971. doi: 10.1038/s41467-023-42780-8 (PMC10693574; doi:10.1038/s41467-023-42780-8)
Supplement: Supplementary file 3 — Description of Additional Supplementary Files [file 41467_2023_42780_MOESM3_ESM.pdf]

Description of Additional Supplementary Files

File Name: Supplementary Data 1
Description: Statistical analysis for Figures 1-6 and Supplementary Figures 1-13.
