# Supplementary material for: Enhanced TARP-γ8-PSD-95 coupling in excitatory neurons contributes to the rapid antidepressant-like action of ketamine in male mice
Source: Nat Commun. 2023 Dec 2;14:7971. doi: 10.1038/s41467-023-42780-8 (PMC10693574; doi:10.1038/s41467-023-42780-8)
Supplement: Supplementary file 5 — Reporting Summary [file 41467_2023_42780_MOESM5_ESM.pdf]

nature portfolio

Corresponding author(s): Fang Wang; Jian-Guo Chen

Last updated by author(s): Oct 10, 2023

Reporting Summary

Nature Portfolio wishes to improve the reproducibility of the work that we publish. This form provides structure for consistency and transparency in reporting. For further information on Nature Portfolio policies, see our Editorial Policies and the Editorial Policy Checklist.

Statistics

For all statistical analyses, confirm that the following items are present in the figure legend, table legend, main text, or Methods section.

n/a	Confirmed	
☐	☒	The exact sample size (*n*) for each experimental group/condition, given as a discrete number and unit of measurement
☐	☒	A statement on whether measurements were taken from distinct samples or whether the same sample was measured repeatedly
☐	☒	The statistical test(s) used AND whether they are one- or two-sided<br>*Only common tests should be described solely by name; describe more complex techniques in the Methods section.*
☒	☐	A description of all covariates tested
☐	☒	A description of any assumptions or corrections, such as tests of normality and adjustment for multiple comparisons
☐	☒	A full description of the statistical parameters including central tendency (e.g. means) or other basic estimates (e.g. regression coefficient) AND variation (e.g. standard deviation) or associated estimates of uncertainty (e.g. confidence intervals)
☐	☒	For null hypothesis testing, the test statistic (e.g. *F*, *t*, *r*) with confidence intervals, effect sizes, degrees of freedom and *P* value noted<br>*Give P values as exact values whenever suitable.*
☒	☐	For Bayesian analysis, information on the choice of priors and Markov chain Monte Carlo settings
☒	☐	For hierarchical and complex designs, identification of the appropriate level for tests and full reporting of outcomes
☒	☐	Estimates of effect sizes (e.g. Cohen's *d*, Pearson's *r*), indicating how they were calculated

Our web collection on statistics for biologists contains articles on many of the points above.

Software and code

Policy information about availability of computer code

Data collection	behavioral recordings were collected with ANY-mazy Version 7.1;Whole-cell patch-clamp recording was recorded with Multiclamp 700B amplifier (Molecular Devices, Sunnyvale, CA, USA) ;Confocal laser scanning microscope (FV1000, Olympus, Tokyo, Japan) was used for brain slice imaging; Western blot images were captured with Li-Cor Odyssey (CLx) imaging system.
Data analysis	Analysis was performed by GraphPad Prism 8.0. The unpaired Student's t-tests, one-way or two-way analysis of variance followed by Dunnett's or Bonferroni's post hoc tests were used appropriately.<br>Axon pClamp 10.3 software was used for electrophysiological analysis.<br>Image Studio Lite (Ver 5.2) software was used for Western blot analysis.

For manuscripts utilizing custom algorithms or software that are central to the research but not yet described in published literature, software must be made available to editors and reviewers. We strongly encourage code deposition in a community repository (e.g. GitHub). See the Nature Portfolio guidelines for submitting code & software for further information.

Data

Policy information about availability of data

All manuscripts must include a data availability statement. This statement should provide the following information, where applicable:

- Accession codes, unique identifiers, or web links for publicly available datasets
- A description of any restrictions on data availability
- For clinical datasets or third party data, please ensure that the statement adheres to our policy

The raw data and blot images generated in this study are provided in the Supplementary Information/Source Data file. Further information generated in this study are available from the corresponding author upon request. Source data are provided with this paper.

Human research participants

Policy information about studies involving human research participants and Sex and Gender in Research.

Reporting on sex and gender	n/a
Population characteristics	n/a
Recruitment	n/a
Ethics oversight	n/a

Note that full information on the approval of the study protocol must also be provided in the manuscript.

Field-specific reporting

Please select the one below that is the best fit for your research. If you are not sure, read the appropriate sections before making your selection.

☒ Life sciences ☐ Behavioural & social sciences ☐ Ecological, evolutionary & environmental sciences

For a reference copy of the document with all sections, see nature.com/documents/nr-reporting-summary-flat.pdf

Life sciences study design

All studies must disclose on these points even when the disclosure is negative.

Sample size	No statistical methods were used to pre-determine sample sizes, but our required sample size was estimated based upon prior experience and all the statistics sample sizes (n) are similar to those reported previously from our group and other labs. Our number of data points have proven to be sufficient evident by the statistical significance.
Data exclusions	In the CSDS model, mice were excluded for poor body condition (e.g. open wounds exceeding 1 cm severe injury). For experiments where virus is infused intracranially, accurate virus injection site was verified after brain collection, and misinjected mice were not analyzed for cellular or behavior data.
Replication	All experiments were repeated at least three times, and similar results were observed.
Randomization	Mice were pooled and then randomly assigned to to each pertinent group.
Blinding	The investigators were blinded to group allocation during data collection and analysis.

Reporting for specific materials, systems and methods

We require information from authors about some types of materials, experimental systems and methods used in many studies. Here, indicate whether each material, system or method listed is relevant to your study. If you are not sure if a list item applies to your research, read the appropriate section before selecting a response.

Materials & experimental systems

n/a	Involved in the study
☐	☒ Antibodies
☒	☐ Eukaryotic cell lines
☒	☐ Palaeontology and archaeology
☐	☒ Animals and other organisms
☒	☐ Clinical data
☒	☐ Dual use research of concern

Methods

n/a	Involved in the study
☒	☐ ChIP-seq
☒	☐ Flow cytometry
☒	☐ MRI-based neuroimaging

Antibodies

Antibodies used

GluA1 (Cat#ab31232; Lot: GR3411557-1; Abcam, Cambridge, UK);
GluA2 (Clone: EP966Y; Cat#ab52932; Lot: GR301181-6; Abcam, Cambridge, UK);
β-actin (Clone: C4; Cat#sc-47778; Lot: G3019; Santa Cruz Biotechnology, Santa Cruz, CA);
PSD-95 (Cat#ab18258; Lot: 1030594-1; Abcam, Cambridge, UK);
CaMKIIα (Clone: 6G9; Cat#50049; Lot: 1; Cell Signaling,Boston, USA);
GAD67(Clone: K-87; Cat#ab26116; Lot: GR3423991-1; Abcam, Cambridge, UK);
Phospho-CaMKII (Clone: D21E4; Cat#12716; Lot: 5; Cell Signaling Technology, Boston, USA);
IRDye® 800CW Goat anti-Mouse IgG Secondary Antibody (Cat# 926-32210; Lot: D01110-03; LI-COR Biosciences, USA);
IRDye® 800CW Goat anti-Rabbit IgG Secondary Antibody (Cat# 926-32211; Lot: D21109-35; LI-COR Biosciences, USA);
Donkey anti-Mouse IgG (H+L) Highly Cross-Adsorbed Secondary Antibody, Alexa Fluor™ 594(Cat# A-21203; Lot: 2352146; ThermoFisher Scientific, Rockford, USA);
Donkey anti-Rabbit IgG (H+L) Highly Cross-Adsorbed Secondary Antibody, Alexa Fluor™ 488 (Cat# A-21206; Lot: 2376850; ThermoFisher Scientific, Rockford, USA).

Validation

All antibodies used in this study were validated by the supplier as follows:
CACNG8 Polyclonal Antibody (PA5-48249, ThermoFisher Scientific, Rockford, USA), https://www.thermofisher.cn/cn/zh/antibody/product/CACNG8-Antibody-Polyclonal/PA5-48249 Suitable for IF, WB;
L-type Ca++ CP γ8 (A-8) (sc-514421, Santa Cruz Biotechnology, Santa Cruz, CA), https://www.scbt.com/p/l-type-ca-cp-gamma8-antibody-a-8?requestFrom=search Suitable for IF, WB,IP;
Anti-Glutamate Receptor 1 (ab31232, Abcam, Cambridge, UK), https://www.abcam.cn/glutamate-receptor-1-ampa-subtype-antibody-ab31232.html Suitable for: WB, IHC-P, IHC-FoFr;
Anti-Ionotropic Glutamate receptor 2 (ab52932, Abcam, Cambridge, UK), https://www.abcam.cn/ionotropic-glutamate-receptor-2-antibody-ep966y-ab52932.html Suitable for: WB;
beta Actin (sc-47778, Santa Cruz Biotechnology, Santa Cruz, CA), https://www.scbt.com/p/beta-actin-antibody-c4?requestFrom=search Suitable for WB, IHC(P), ELISA;
Anti-PSD95 (ab18258, Abcam, Cambridge, UK). https://www.abcam.cn/psd95-antibody-synaptic-marker-ab18258.html Suitable for ICC/IF, WB, IHC-P;
CaMKII-α (6G9) Mouse mAb (Cell Signaling,Danvers, USA)https://www.cellsignal.cn/products/primary-antibodies/camkii-a-6g9-mouse-mab/50049?_=1671783933445&Ntt=50049&tahead=true Suitable for IF, WB,IHC;
Anti-GAD67 (ab26116, Abcam, Cambridge,UK) https://www.abcam.cn/gad67-antibody-k-87-ab26116.html Suitable for: ICC/IF, Flow Cyt, IHC-P, WB;
Anti-Phospho-CaMKII (Thr286) (12716, Cell Signaling Technology, Boston, USA) https://www.cellsignal.cn/products/primary-antibodies/phospho-camkii-thr286-d21e4-rabbit-mab/12716?site-search-type=Products&N=4294956287&Ntt=12716&fromPage=plp&_requestid=6794023 Suitable for: WB;
IRDye® 800CW Goat anti-Mouse IgG Secondary Antibody https://www.licor.com/bio/reagents/irdye-800cw-goat-anti-mouse-igg-secondary-antibody
IRDye® 800CW Goat anti-Rabbit IgG Secondary Antibody https://www.licor.com/bio/reagents/irdye-800cw-goat-anti-rabbit-igg-secondary-antibody
Donkey anti-Mouse IgG (H+L) Highly Cross-Adsorbed Secondary Antibody, Alexa Fluor™ 594 https://www.thermofisher.cn/cn/zh/antibody/product/Donkey-anti-Mouse-IgG-H-L-Highly-Cross-Adsorbed-Secondary-Antibody-Polyclonal/A-21203
Donkey anti-Rabbit IgG (H+L) Highly Cross-Adsorbed Secondary Antibody, Alexa Fluor™ 488 https://www.thermofisher.cn/cn/zh/antibody/product/Donkey-anti-Rabbit-IgG-H-L-Highly-Cross-Adsorbed-Secondary-Antibody-Polyclonal/A-21206

Eukaryotic cell lines

Policy information about cell lines and Sex and Gender in Research

Cell line source(s)	n/a
Authentication	n/a
Mycoplasma contamination	n/a
Commonly misidentified lines (See ICLAC register)	n/a

Palaeontology and Archaeology

Specimen provenance	n/a
Specimen deposition	n/a
Dating methods	n/a

☐ Tick this box to confirm that the raw and calibrated dates are available in the paper or in Supplementary Information.

Ethics oversight	*Identify the organization(s) that approved or provided guidance on the study protocol, OR state that no ethical approval or guidance was required and explain why not.*

Note that full information on the approval of the study protocol must also be provided in the manuscript.

Animals and other research organisms

Policy information about studies involving animals; ARRIVE guidelines recommended for reporting animal research, and Sex and Gender in Research

Laboratory animals	Adult male C57BL/6J mice (7 weeks of age, 18-22 g) were obtained from Hunan SJA Laboratory Animal Co., Ltd (Changsha, Hunan, China). CD-1 mice (< 4 months of age) provided by Beijing Vital River Laboratory Animal Technology Co., Ltd (Beijing, China) were retired breeders and singly housed throughout. All animals were housed under 12 h:12 h light/dark cycle with consistent ambient temperature (21-23 °C) and humidity (50% ± 5%) and administered food and water ad libitum.
Wild animals	No wild animals was used
Reporting on sex	male mice were used in the experiments.
Field-collected samples	This study did not involve samples collected from the fields.
Ethics oversight	All animal procedures were in accordance with the institutional Animal Care and Use Committee of Huazhong University of Science and Technology.

Note that full information on the approval of the study protocol must also be provided in the manuscript.

Clinical data

Policy information about clinical studies

All manuscripts should comply with the ICMJE guidelines for publication of clinical research and a completed CONSORT checklist must be included with all submissions.

Clinical trial registration	n/a
Study protocol	n/a
Data collection	n/a
Outcomes	n/a

Dual use research of concern

Policy information about dual use research of concern

Hazards

Could the accidental, deliberate or reckless misuse of agents or technologies generated in the work, or the application of information presented in the manuscript, pose a threat to:

No	Yes	
☒	☐	Public health
☒	☐	National security
☒	☐	Crops and/or livestock
☒	☐	Ecosystems
☒	☐	Any other significant area

Experiments of concern

Does the work involve any of these experiments of concern:

No	Yes	
☒	☐	Demonstrate how to render a vaccine ineffective
☒	☐	Confer resistance to therapeutically useful antibiotics or antiviral agents
☒	☐	Enhance the virulence of a pathogen or render a nonpathogen virulent
☒	☐	Increase transmissibility of a pathogen
☒	☐	Alter the host range of a pathogen
☒	☐	Enable evasion of diagnostic/detection modalities
☒	☐	Enable the weaponization of a biological agent or toxin
☒	☐	Any other potentially harmful combination of experiments and agents

ChIP-seq

Data deposition

☐ Confirm that both raw and final processed data have been deposited in a public database such as GEO.

☐ Confirm that you have deposited or provided access to graph files (e.g. BED files) for the called peaks.

Data access links<br>*May remain private before publication.*	*For "Initial submission" or "Revised version" documents, provide reviewer access links. For your "Final submission" document, provide a link to the deposited data.*
Files in database submission	*Provide a list of all files available in the database submission.*
Genome browser session<br>(e.g. UCSC)	*Provide a link to an anonymized genome browser session for "Initial submission" and "Revised version" documents only, to enable peer review. Write "no longer applicable" for "Final submission" documents.*

Methodology

Replicates	*Describe the experimental replicates, specifying number, type and replicate agreement.*
Sequencing depth	*Describe the sequencing depth for each experiment, providing the total number of reads, uniquely mapped reads, length of reads and whether they were paired- or single-end.*
Antibodies	*Describe the antibodies used for the ChIP-seq experiments; as applicable, provide supplier name, catalog number, clone name, and lot number.*
Peak calling parameters	*Specify the command line program and parameters used for read mapping and peak calling, including the ChIP, control and index files used.*
Data quality	*Describe the methods used to ensure data quality in full detail, including how many peaks are at FDR 5% and above 5-fold enrichment.*
Software	*Describe the software used to collect and analyze the ChIP-seq data. For custom code that has been deposited into a community repository, provide accession details.*

Flow Cytometry

Plots

Confirm that:

☐ The axis labels state the marker and fluorochrome used (e.g. CD4-FITC).

☐ The axis scales are clearly visible. Include numbers along axes only for bottom left plot of group (a 'group' is an analysis of identical markers).

☐ All plots are contour plots with outliers or pseudocolor plots.

☐ A numerical value for number of cells or percentage (with statistics) is provided.

Methodology

Sample preparation	*Describe the sample preparation, detailing the biological source of the cells and any tissue processing steps used.*
Instrument	*Identify the instrument used for data collection, specifying make and model number.*

Software	*Describe the software used to collect and analyze the flow cytometry data. For custom code that has been deposited into a community repository, provide accession details.*
Cell population abundance	*Describe the abundance of the relevant cell populations within post-sort fractions, providing details on the purity of the samples and how it was determined.*
Gating strategy	*Describe the gating strategy used for all relevant experiments, specifying the preliminary FSC/SSC gates of the starting cell population, indicating where boundaries between "positive" and "negative" staining cell populations are defined.*

☐ Tick this box to confirm that a figure exemplifying the gating strategy is provided in the Supplementary Information.

Magnetic resonance imaging

Experimental design

Design type	*Indicate task or resting state; event-related or block design.*
Design specifications	*Specify the number of blocks, trials or experimental units per session and/or subject, and specify the length of each trial or block (if trials are blocked) and interval between trials.*
Behavioral performance measures	*State number and/or type of variables recorded (e.g. correct button press, response time) and what statistics were used to establish that the subjects were performing the task as expected (e.g. mean, range, and/or standard deviation across subjects).*

Acquisition

Imaging type(s)	*Specify: functional, structural, diffusion, perfusion.*
Field strength	*Specify in Tesla*
Sequence & imaging parameters	*Specify the pulse sequence type (gradient echo, spin echo, etc.), imaging type (EPI, spiral, etc.), field of view, matrix size, slice thickness, orientation and TE/TR/flip angle.*
Area of acquisition	*State whether a whole brain scan was used OR define the area of acquisition, describing how the region was determined.*

Diffusion MRI ☐ Used ☐ Not used

Preprocessing

Preprocessing software	*Provide detail on software version and revision number and on specific parameters (model/functions, brain extraction, segmentation, smoothing kernel size, etc.).*
Normalization	*If data were normalized/standardized, describe the approach(es): specify linear or non-linear and define image types used for transformation OR indicate that data were not normalized and explain rationale for lack of normalization.*
Normalization template	*Describe the template used for normalization/transformation, specifying subject space or group standardized space (e.g. original Talairach, MNI305, ICBM152) OR indicate that the data were not normalized.*
Noise and artifact removal	*Describe your procedure(s) for artifact and structured noise removal, specifying motion parameters, tissue signals and physiological signals (heart rate, respiration).*
Volume censoring	*Define your software and/or method and criteria for volume censoring, and state the extent of such censoring.*

Statistical modeling & inference

Model type and settings	*Specify type (mass univariate, multivariate, RSA, predictive, etc.) and describe essential details of the model at the first and second levels (e.g. fixed, random or mixed effects; drift or auto-correlation).*
Effect(s) tested	*Define precise effect in terms of the task or stimulus conditions instead of psychological concepts and indicate whether ANOVA or factorial designs were used.*

Specify type of analysis: ☐ Whole brain ☐ ROI-based ☐ Both

Statistic type for inference (See Eklund et al. 2016)	*Specify voxel-wise or cluster-wise and report all relevant parameters for cluster-wise methods.*
Correction	*Describe the type of correction and how it is obtained for multiple comparisons (e.g. FWE, FDR, permutation or Monte Carlo).*

Models & analysis

n/a	Involved in the study
☐	☐ Functional and/or effective connectivity
☐	☐ Graph analysis
☐	☐ Multivariate modeling or predictive analysis

Functional and/or effective connectivity

Report the measures of dependence used and the model details (e.g. Pearson correlation, partial correlation, mutual information).

Graph analysis

Report the dependent variable and connectivity measure, specifying weighted graph or binarized graph, subject- or group-level, and the global and/or node summaries used (e.g. clustering coefficient, efficiency, etc.).

Multivariate modeling and predictive analysis

Specify independent variables, features extraction and dimension reduction, model, training and evaluation metrics.
